# Supplementary material for: Biophysical feedback of global forest fires on surface temperature
Source: Nat Commun. 2019 Jan 15;10:214. doi: 10.1038/s41467-018-08237-z (PMC6333928; doi:10.1038/s41467-018-08237-z)
Supplement: Supplementary file 1 — Supplementary Information [file 41467_2018_8237_MOESM1_ESM.pdf]

Supplementary information for

Biophysical feedback of global forest fires on surface temperature

Liu et al.

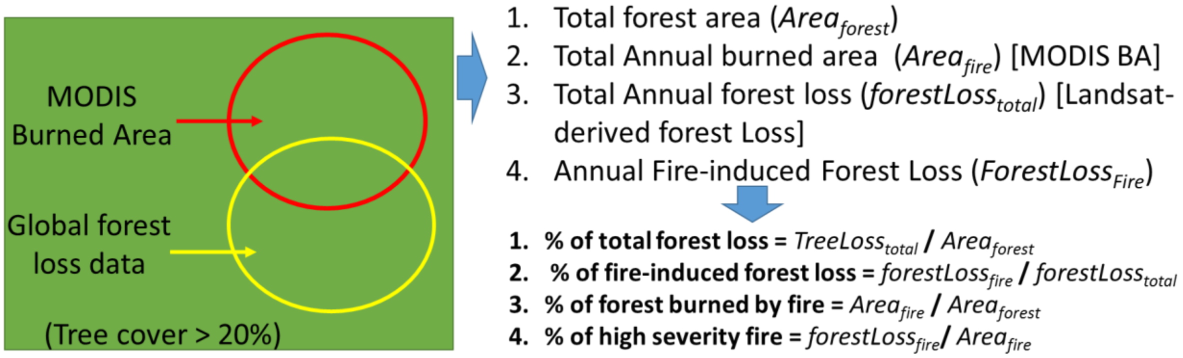

Supplementary Figure 1. Schematic illustration demonstrating how to quantify global fire-induced forest loss.

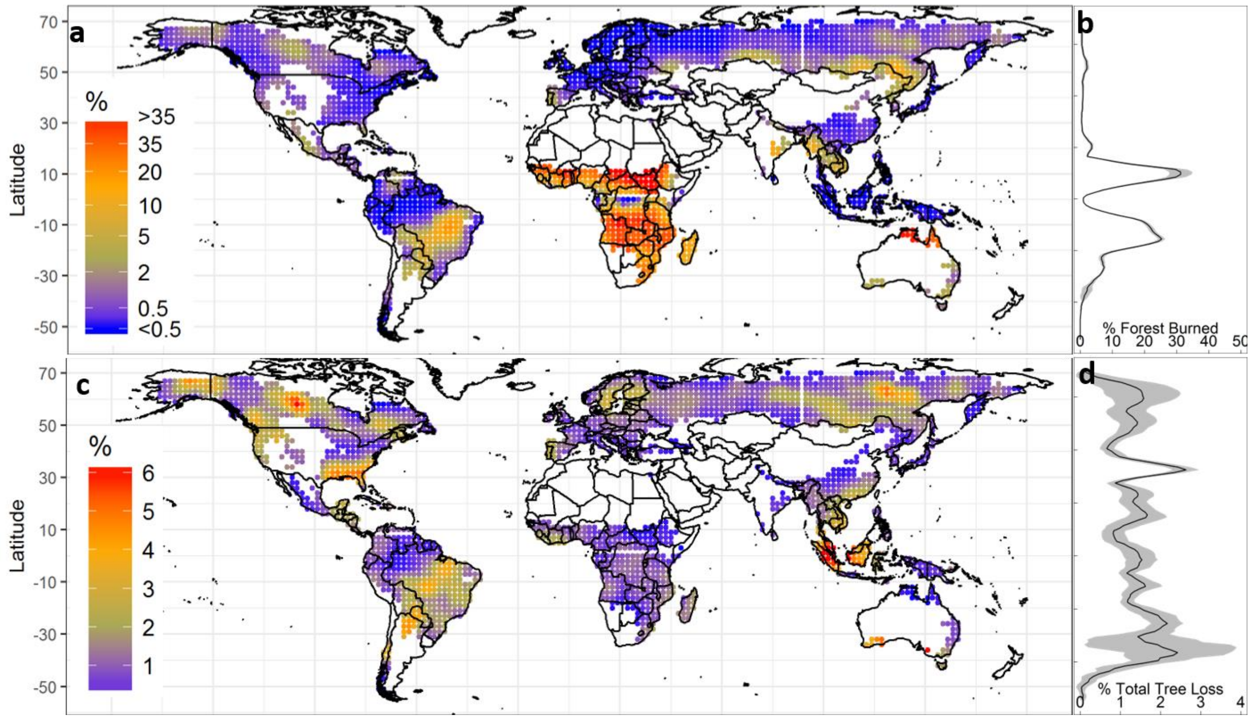

Supplementary Figure 2. The spatial patterns of percent of (a) annual burned area, and (c) total tree loss between 2003 and 2014. (b) and (d) are the latitudinal summaries of (a) and (c), respectively. Points are spaced 2 x 2 degree in both latitude and longitude, and smoothed by 4 x 4 degree moving windows. In b and d, shaded areas are the mean ± one standard deviation. Figures a and c were created in the R environment for statistical computing and graphics (https://www.r-project.org/).

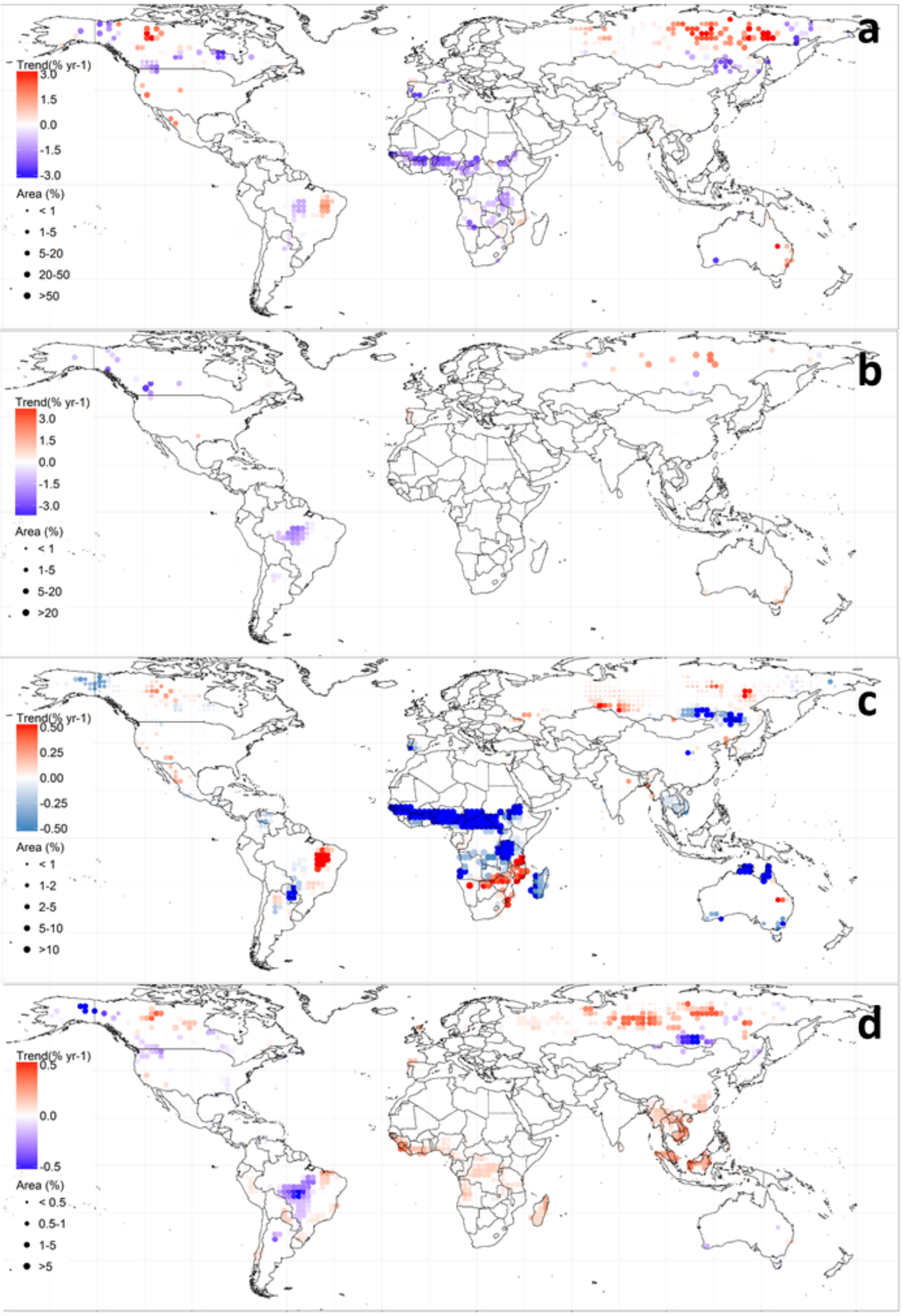

Supplementary Figure 3. The spatial patterns of (a) percent of fire-induced tree cover loss and its trend, (b) percent of high severity fire and its trend, (c) percent of burned forest and its trend, (d) and total tree loss and its trend. The symbol size indicates magnitude and the color specifies trend direction (red = positive, blue = negative). Points are spaced 2 x 2 degree in both latitude and longitude, and smoothed by 4 x 4 degree moving windows. Only significant ($p < 0.1$) trends from 2003-2014 were plotted. Figures a-b were created in the R environment for statistical computing and graphics (https://www.r-project.org/).

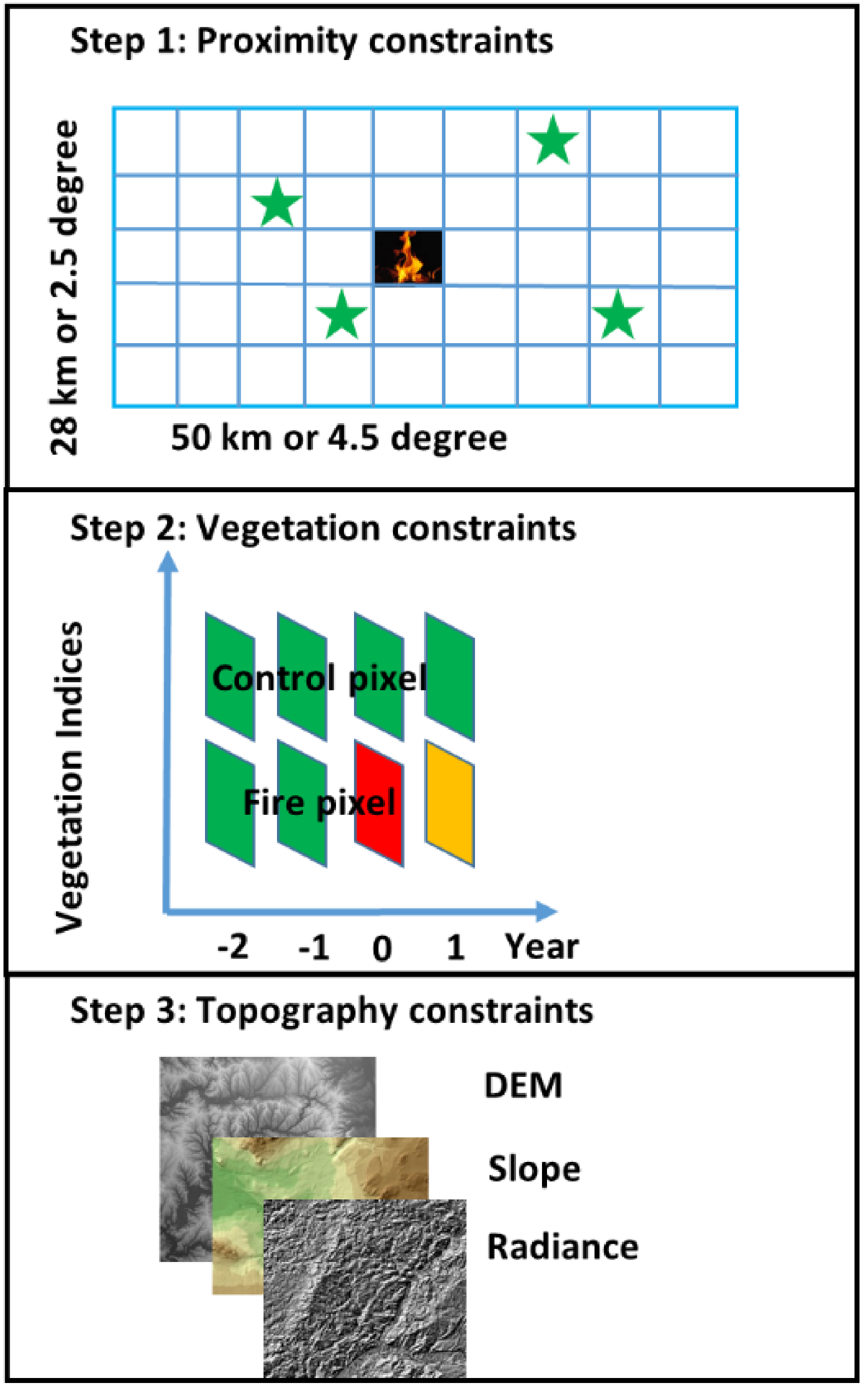

Supplementary Figure 4. Schematic illustration showing the sampling procedure for our space-for-time approach.

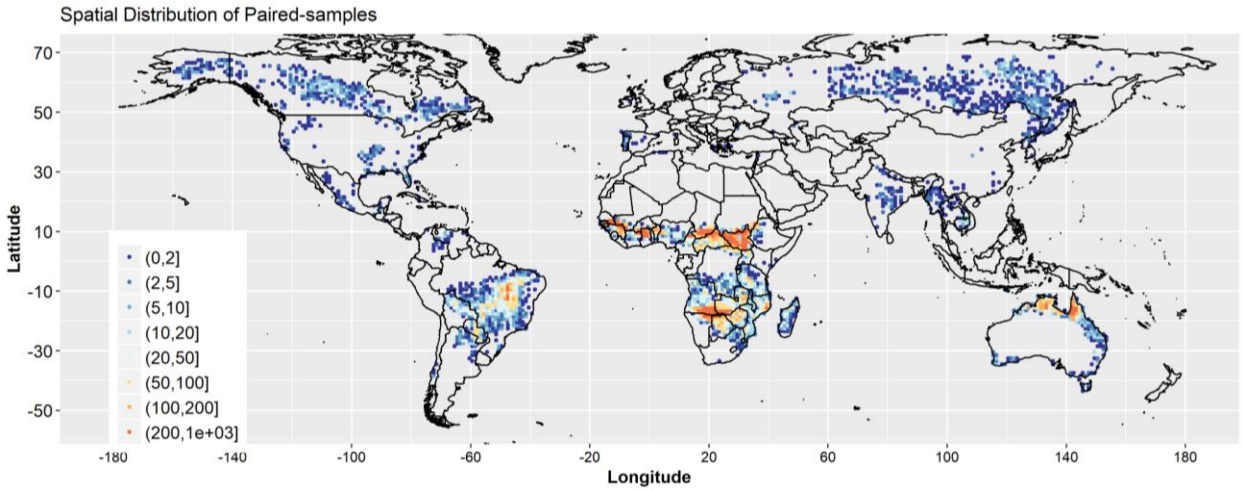

Supplementary Figure 5. The spatial distribution of paired fire-control samples within each 1° pixel. Figure was created in the R environment for statistical computing and graphics (https://www.r-project.org/).

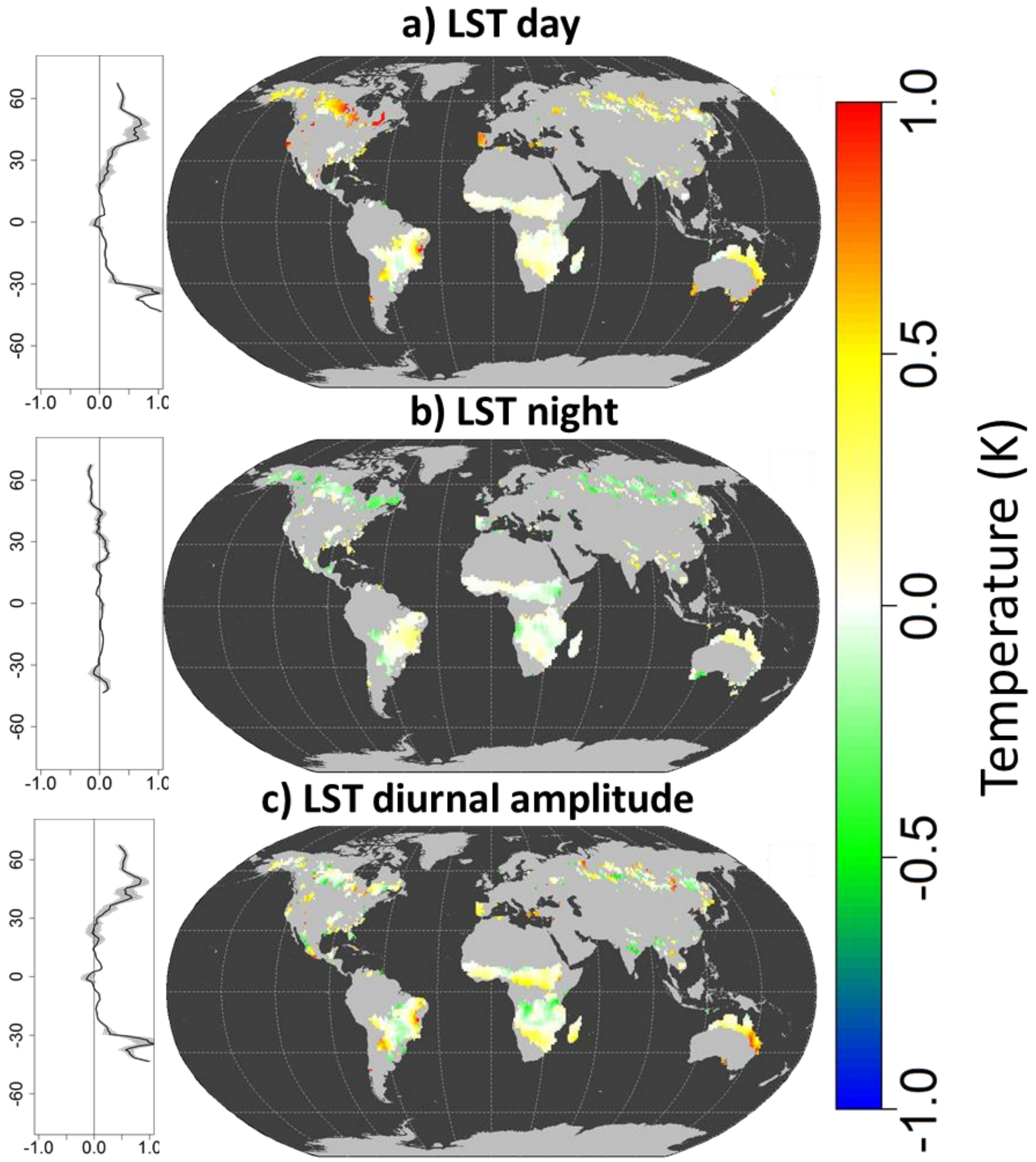

Supplementary Figure 6. The mean annual biophysical effects of fire-induced forest loss on land surface radiometric temperatures (LST) for (a) daytime, (b) nighttime, and (c) diurnal amplitude (defined as the difference between a and b). Shaded areas are the mean ± one standard deviation. Similar to deforestation, we found that fire-induced forest loss also caused a nighttime cooling effect in northern high latitudes, probably due to reduce the efficiency of bringing heat from aloft to the surface during stable atmospheric conditions from loss of forest cover [1,2]. Figures a-c were created in the R environment for statistical computing and graphics (https://www.r-project.org/).

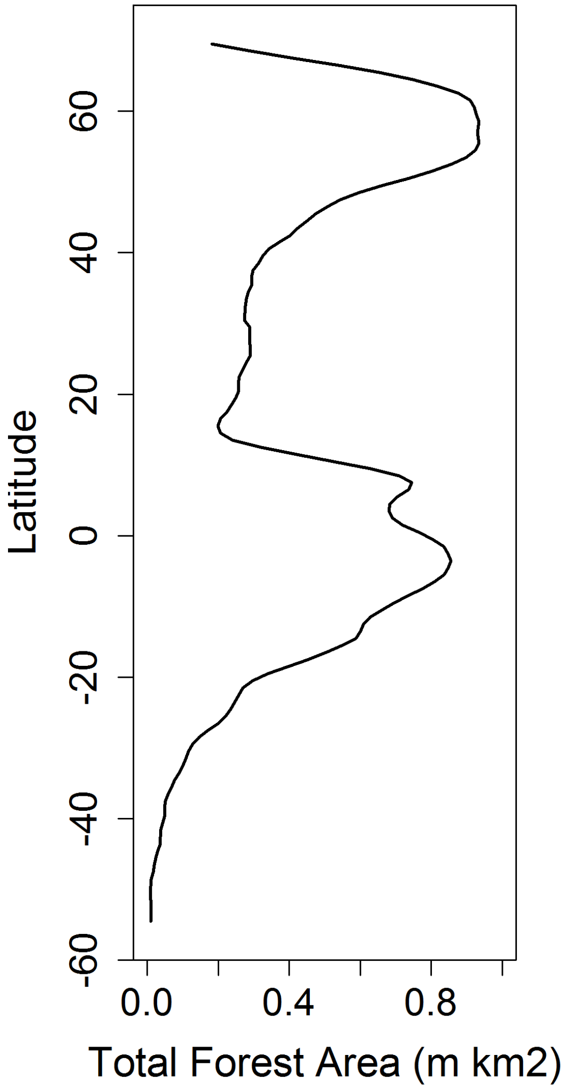

Supplementary Figure 7. Latitudinal summary of total forest area within 1° latitude based on MODIS land cover data.

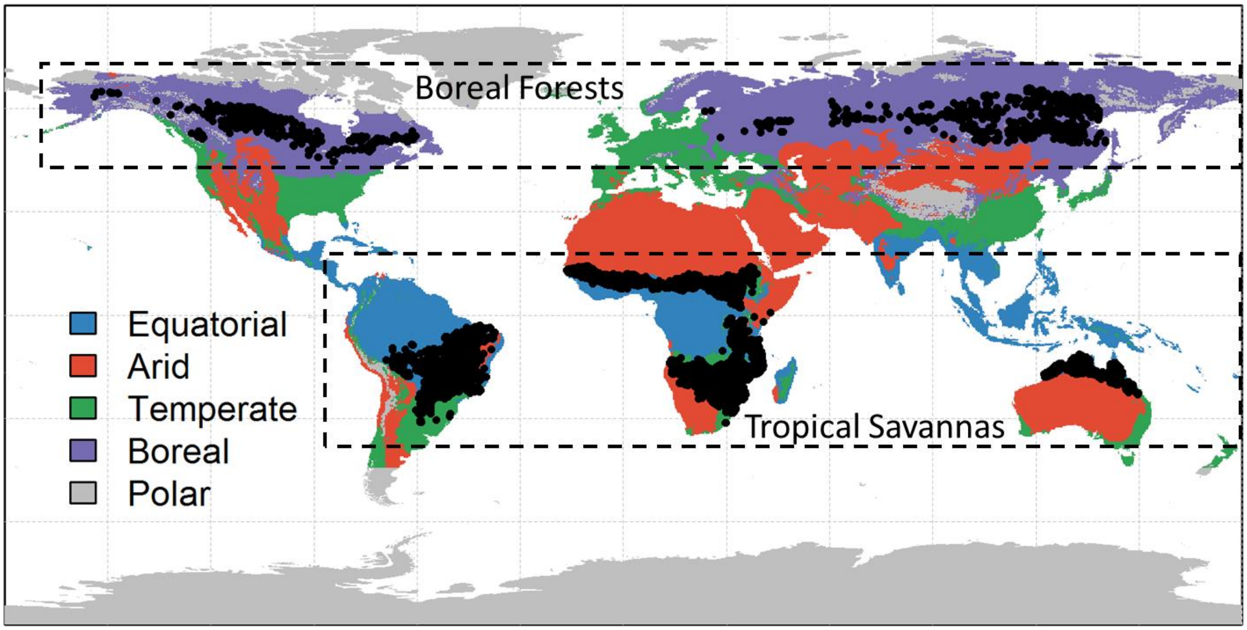

Supplementary Figure 8. The distribution of paired fire-control samples (black points) within boreal forests and tropical savannas regions, overlaid on the Köppen-Geiger climate classification map. The rectangles are descriptive only, and not used to define boreal forests and tropical savannas in this analysis. Figure was created in the R environment for statistical computing and graphics (https://www.r-project.org/).

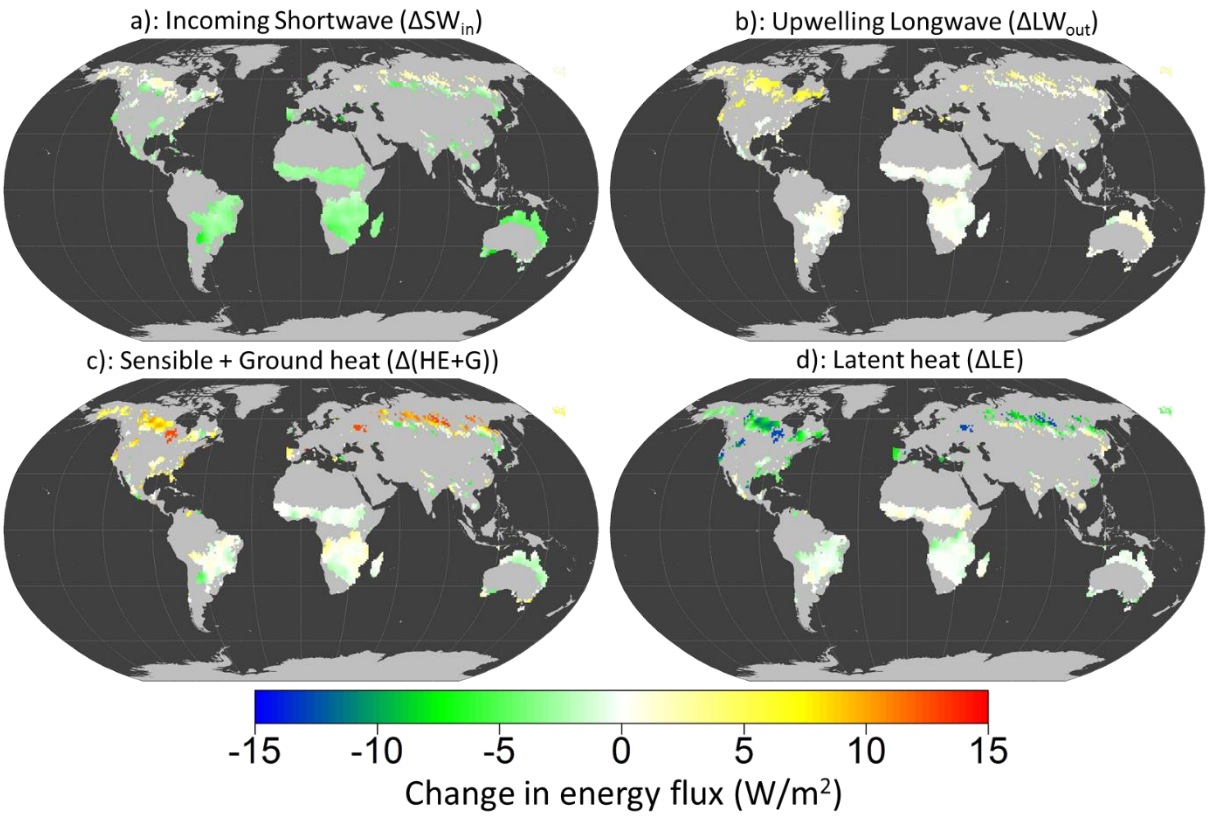

Supplementary Figure 9. Average summertime (JJA) changes in (a) incoming shortwave radiation (ΔSW_{in}), (b) longwave emitted radiation (ΔLW_{out}), (c) the latent heat flux (ΔLE), and (d) the combination of sensible and ground heat fluxes ($\Delta(HE+G)$). Figures a-b were created in the R environment for statistical computing and graphics (https://www.r-project.org/).

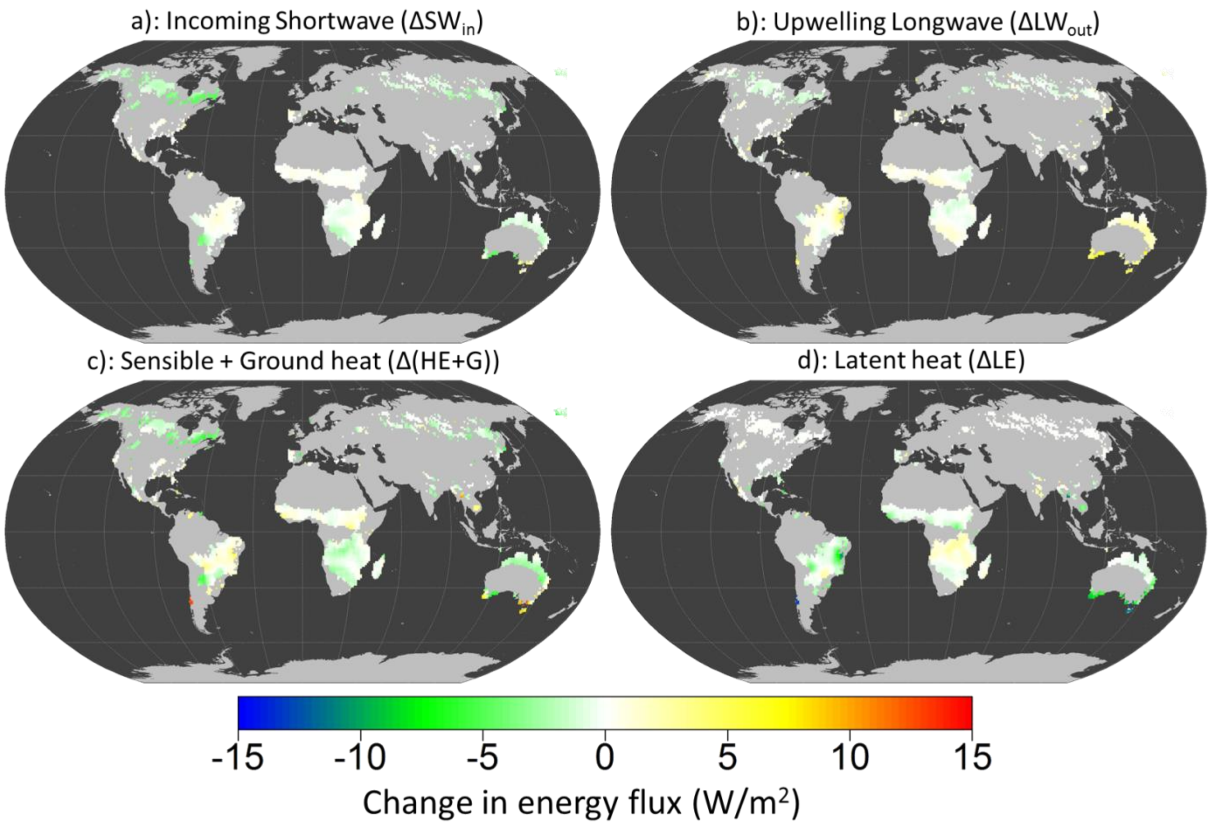

Supplementary Figure 10. Average wintertime (DJF) changes in (a) incoming shortwave radiation (ΔSW_{in}), (b) longwave emitted radiation (ΔLW_{out}), (c) the latent heat flux (ΔLE), and (d) the combination of sensible and ground heat fluxes ($\Delta(HE+G)$). Figures a-b were created in the R environment for statistical computing and graphics (https://www.r-project.org/).

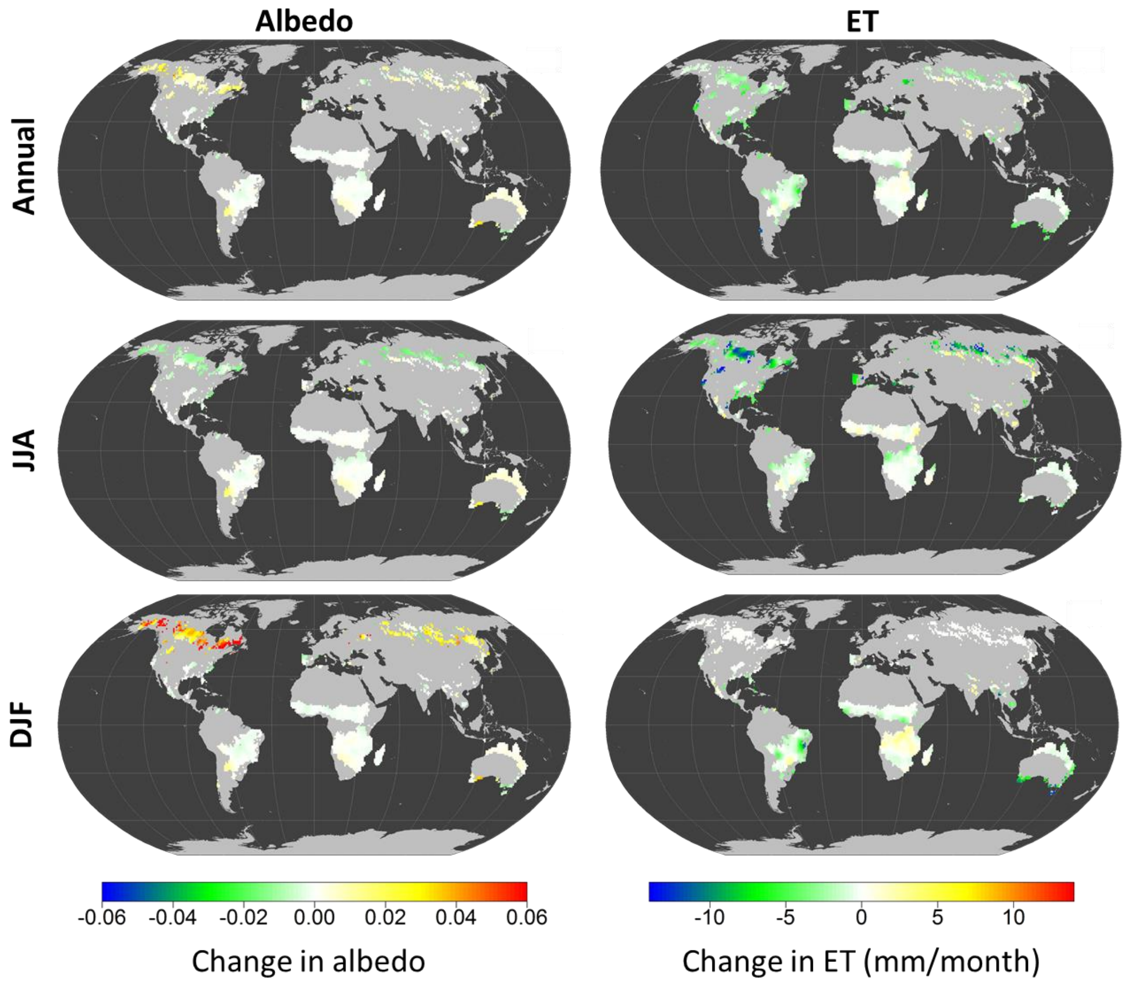

Supplementary Figure 11. Average annual (top), summer (JJA), and winter (DJF) changes in albedo (left) and evapotranspiration (right). Figures were created in the R environment for statistical computing and graphics (https://www.r-project.org/).

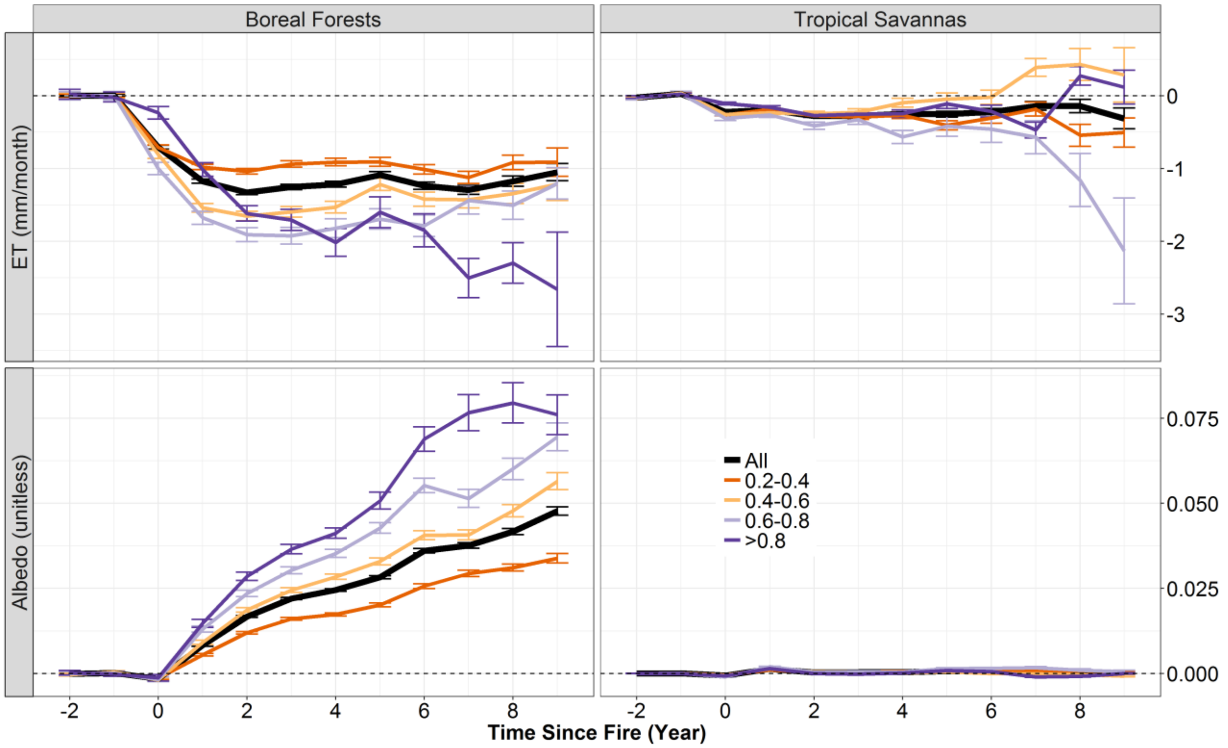

Supplementary Figure 12. Time series plots of evapotranspiration and albedo for tropical savannas and boreal forests, stratified by percent of area burned within each 0.05-degree grid (i.e., All, 0.2-0.4, 0.4-0.6, 0.6-0.8, > 0.8). Error bars are one standard deviation.

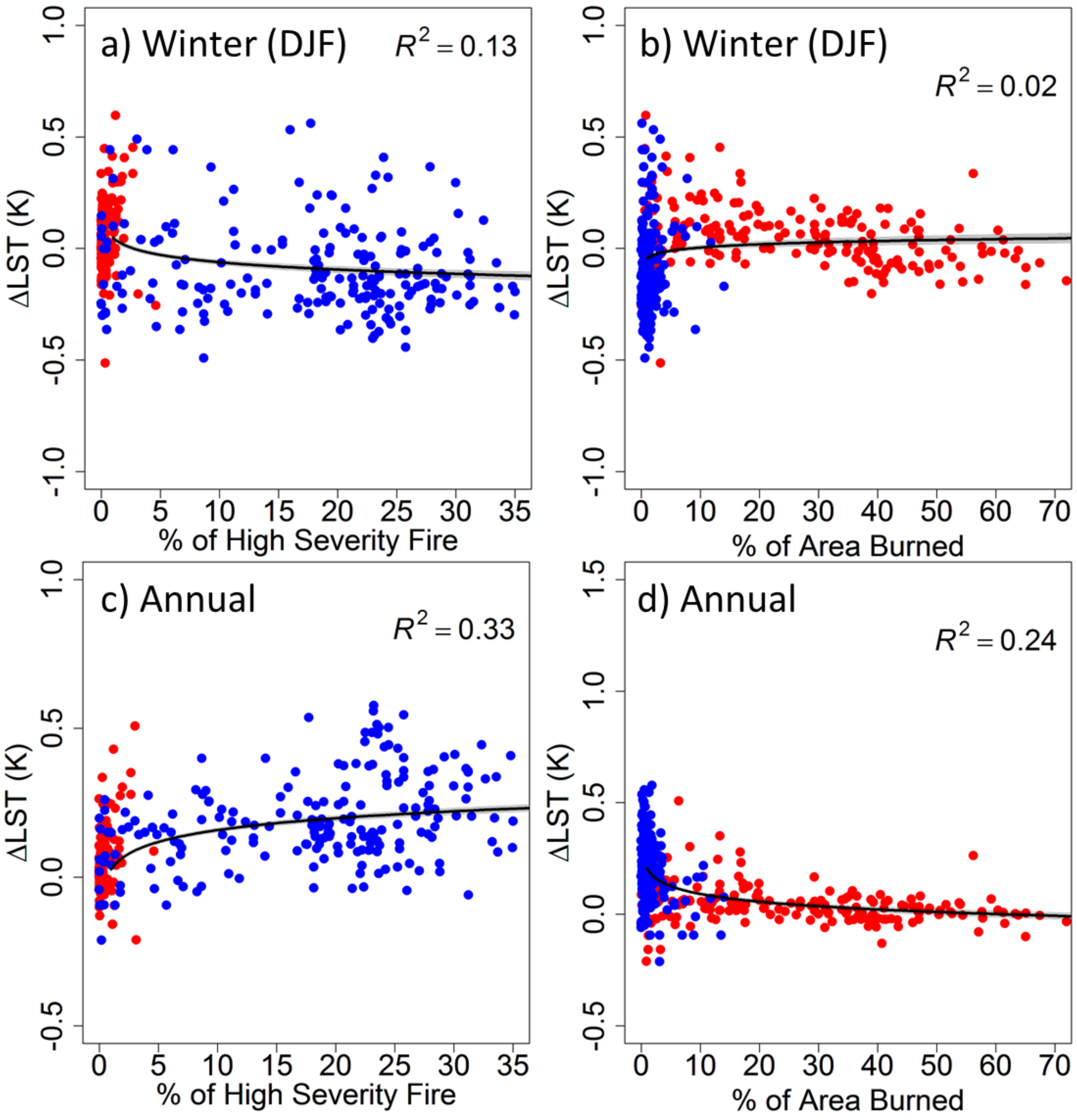

Supplementary Figure 13. Effects of fire severity (a, c) and burned area (b, d) on winter (a, b) and annual (c, d) surface temperatures. Points represent summaries over 2 × 2 degree windows. Blue points show results from boreal forests while red points show results from the tropical savannas. Climate regions are defined by the Köppen-Geiger climate classification system (see Supplementary Figure 8).

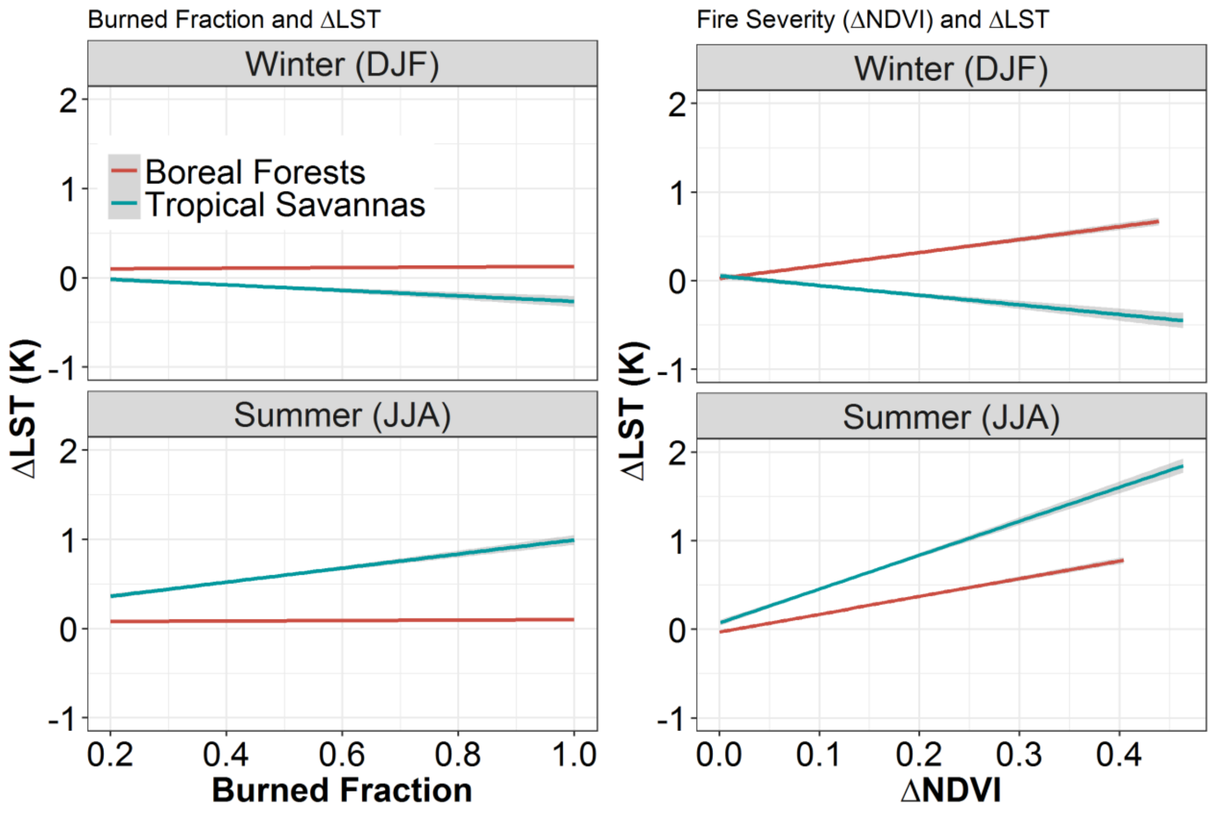

Supplementary Figure 14. Effects of burned area (percent of area burned) and fire severity (ΔNDVI) on ΔLST at the pixel level (0.05˚) for boreal forests and tropical savannas (see Supplementary Figure 8).

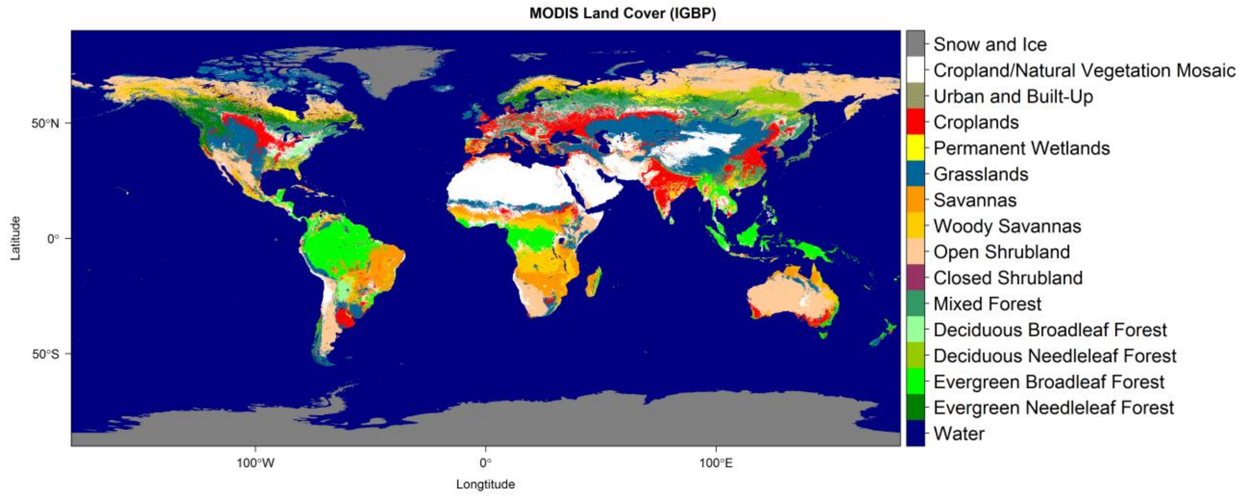

Supplementary Figure 15. MODIS land cover (IGBP classification) map. Figure was created in the R environment for statistical computing and graphics (https://www.r-project.org/).

Supplementary Table 1: zonal statistics for ΔLST, biophysical processes and resultant change in energy flux. Numbers are reported as mean ± S.D.

Region	ΔLST			Δα(unitless)			ΔSW_{in} (W m^{-2}) (=SW↓×(1 - Δα))		
	Winter	Summer	Annual	Winter	Summer	Annual	Winter	Summer	Annual
**Northern High Latitude (>45N)**	-0.164 ± 0058	0.664 ± 0.038	0.198 ± 0.044	0.033 ± 0.0038	-0.0096 ± 0.0031	0.0068 ± 0.0015	-2.05±0.95	2.97±0.85	0.28±0.41
**Northern Mid-Latitude (20N-45N)**	0.111 ± 0.023	0.188 ± 0.144	0.155± 0.062	0.00023 ± 0.0036	-0.00066± 0.00084	-0.00039± 0.00081	0.041±0.41	0.21±0.27	0.14±0.16
**Low latitude (20S - 20N)**	0.0285± 0.062	0.037± 0.059	0.039± 0.045	-0.00020 ± 0.00072	-0.000071± 0.0015	-0.000031± 0.00094	0.059±0.24	0.042±0.38	0.023±0.26
**Southern Mid-Latitude (20N-45N)**	0.507± 0.270	0.110± 0.056	0.288± 0.14	0.00089 ± 0.0041	-0.00038± 0.0054	0.00037± 0.0048	-0.33 ±1.45	-0.12 ±0.77	-0.25 ±1.13

Supplementary Table 1 *continued*

Region	ΔET (mm month-1)			ΔLE (W m^{-2}) (= ΔET×28.94, ET in mm/day)			ΔHE (W m^{-2}) (= ΔSW_{in} - ΔLE)		
	Winter	Summer	Annual	Winter	Summer	Annual	Winter	Summer	Annual
**Northern High Latitude (>45N)**	0.16 ±0.066	-8.19 ±1.13	-2.13±0.35	0.15±0.063	-7.72±1.07	-2.00±0.33	-2.19±0.97	10.69±1.25	2.78±0.48
**Northern Mid-Latitude (20N-45N)**	-0.38±0.32	-3.47±1.99	-2.15±0.91	-0.39±0.31	-3.28±1.87	-2.04±0.86	0.44±0.63	3.51±1.83	2.21±0.89
**Low latitude (20S - 20N)**	-0.14±0.59	-0.79±0.44	-0.53±0.44	-0.14±0.58	-0.74±0.42	-0.51±0.42	0.19±0.59	0.73±0.75	0.53±0.51
**Southern Mid-Latitude (20N-45N)**	-5.87±4.22	-0.81±0.61	-2.75±1.79	-5.68±4.08	-0.76±0.58	-2.64±1.72	5.34±5.21	0.64±1.14	2.39±2.58

Supplementary References

1 Lee, X. *et al.* Observed increase in local cooling effect of deforestation at higher latitudes. *Nature* **479**, 384-387, doi:10.1038/nature10588 (2011).

2 Schultz, N. M., Lawrence, P. J. & Lee, X. H. Global satellite data highlights the diurnal asymmetry of the surface temperature response to deforestation. *Journal of Geophysical Research-Biogeosciences* **122**, 903-917, doi:10.1002/2016jg003653 (2017).
